# Assessing Nasal Nitric Oxide in Allergic Rhinitis: A Controversial Biomarker

**DOI:** 10.3390/medicina61030516

**Published:** 2025-03-17

**Authors:** Natalia Louca, Despina Damianou, Nektaria Kostea, Panayiotis Kouis, Panayiotis Yiallouros, Constantinos Pitsios

**Affiliations:** Medical School, University of Cyprus, P.O. Box 20537, 1678 Nicosia, Cyprus; louca.natalia@ucy.ac.cy (N.L.); damianou.despina@ucy.ac.cy (D.D.); kostea.nektaria@ucy.ac.cy (N.K.); kouis.panayiotis@ucy.ac.cy (P.K.); yiallouros.panayiotis@ucy.ac.cy (P.Y.)

**Keywords:** allergic rhinitis, nonallergic rhinitis, nasal NO, atopy, allergens

## Abstract

*Background and objectives:* Increased levels of nitric oxide (NO) are produced in various inflammatory diseases like allergic asthma. Fractional exhaled NO has been studied as a biomarker of type 2 inflammation in asthma, while the use of nasal NO (nNO) as a diagnostic tool for allergic rhinitis (AR) is less established. In the present study, we investigated nNO as a potential biomarker for differentiating AR from nonallergic rhinitis (NAR). *Materials and methods:* Medical students were invited to complete a questionnaire on rhinitis symptoms. Individuals who reported nasal symptoms were invited to participate in the clinical phase of the study, which included considering the patient’s medical history, clinical examination, skin-prick tests (SPTs) for the 14 most relevant allergens in the region, and nNO measurement using the NIOX VERO portable nitric oxide analyzer. Informed consent was obtained at each stage of recruitment and clinical assessment. *Results:* Overall, 62 out of 122 volunteers recruited reported rhinitis symptoms and were investigated further with nNO measurements and SPTs. In total, 39 had SPT-confirmed AR, while 23 were classified as NAR subjects. Both nNO measurements and SPTs were performed on the same day, during the pollen season. The comparison of mean nNO concentrations (830 ± 247 ppb and 851 ± 373 in AR and NAR groups, respectively) showed no statistically significant difference. *Conclusions:* we concluded that nNO is not a reliable independent biomarker in the diagnosis of AR.

## 1. Introduction

Rhinitis is a common inflammatory disorder affecting both adults and children, resulting in a significant economic burden. The global prevalence of unspecified rhinitis is about 30% and 18.1% of allergic rhinitis (AR) [1]. AR symptoms can negatively affect the patients’ quality of life and compromise school or work performance. Additionally, AR is a risk factor for other diseases, including asthma [2]. There are distinct phenotypes of rhinitis, established based on the clinical presentation and underlying pathophysiological mechanisms [3]. Differentiating between AR and nonallergic rhinitis (NAR) diagnostically enables the implementation of personalized medicine [3]. AR is an IgE-mediated disorder, and its diagnosis involves physical examination via in vivo (skin prick tests) and in vitro (serum-specific IgE) tests in combination with consideration of the patient’s medical history [4].

Nitric oxide (NO) is the smallest cellular signaling molecule known. It is produced by NO synthase, which uses L-arginine and molecular oxygen as substrates [5]. NO participates in various physiological functions, such as neurotransmission, vasodilation, circulatory system regulation, and immune response [6]. NO synthase exists in three isoforms, the neural NOS (nNOS), the endothelial NOS (eNOS), and the inducible NOS (iNOS) [6]. nNOS and eNOS are constitutively expressed in the body and their activities are controlled by the intracellular calcium levels. nNOS and eNOS produce very small amounts of NO for a short period due to their Ca^2+^ dependency. In contrast, iNOS is expressed when the cell is stimulated, mostly by proinflammatory cytokines, does not depend on Ca^2+^, and upon induction tends to generate a considerable amount of NO, which lasts for a long time [5,6].

Patients with inflammatory diseases demonstrate increased levels of NO due to overproduction by the iNOS enzyme. Fractional exhaled NO (FeNO) is produced in lower airways and is used as a biomarker of high specificity for type 2 inflammation in asthma and as a predictive tool of asthma responses to treatment with corticosteroids [7]. Nasal NO (nNO) is measured in the upper airway and has been used as an additional tool for the diagnosis of primary ciliary dyskinesia (PCD) [8,9]. However, nNO measurement is not yet an established diagnostic tool for AR [10].

Based on the established use of FeNO for phenotyping small airway disease, the measurement of nNO may be a helpful non-invasive method for phenotyping chronic rhinitis. The potential use of nNO as a biomarker, representing the degree of allergic inflammation in the nasal cavities, could be very useful in the diagnosis of AR and for the follow-up of anti-inflammatory treatment. Previous studies, comparing nNO levels in patients with AR and NAR, have shown controversial results so far, with some of them reporting an increase, others revealing a decrease, and some showing similar nNO levels among patients with AR and NAR [11,12,13].

The “Prevention of Allergic Rhinitis in Cyprus” (PARC) project is an initiative aiming to diagnose and follow up AR in university students. Within the frame of the PARC study, we aimed to evaluate nNO as a biomarker of AR, comparing nNO levels between individuals with AR or NAR. We also aimed to detect any difference in nNO levels between AR subjects with perennial or seasonal allergen sensitizations, as well as the effect of the sensitizations’ number (positive skin prick test, SPT) on nNO levels.

## 2. Materials and Methods

### 2.1. Study Population

The study population consisted of students from the faculty of Medicine of the University of Cyprus aged 18–25 years old. The research protocol was approved by the Cyprus National Bioethics Committee (protocol code 2018/41) and this study was part of the PARC project. All volunteers provided written informed consent before their enrollment in the study. The process of recruitment was carried out between February and March 2023.

Students were screened with the use of a questionnaire and were asked if nasal symptoms (nasal congestion, rhinorrhea, sneezing, and nasal itching) appeared during the last 12 months, aside from days with an occurring respiratory infection. Participants that had at least one “bothering” rhinitis symptom were further asked for the symptoms of conjunctivitis, seasonal exacerbation, symptoms related to house dust or pets, the frequency (duration more than 4 days per week and/or more than a month per year), the use of any nasal spray, former medical diagnosis of AR, asthma, or atopic dermatitis, and family history of atopic dermatitis/AR/conjunctivitis/asthma.

Students with anamnesis of rhinitis-related symptoms underwent further atopy screening and nNO measurements.

### 2.2. Assessment of Allergy

In order to assess the atopic status of the volunteers who reported rhinitis symptoms, SPTs were carried out following a standard procedure and using a panel of commercial extracts (Allergy Therapeutics, Worthing, UK) of the most relevant airborne allergens in Cyprus; *Olea europea*, *Cupressus sempervirens*, *Phleum pratense*, *Zea mais, Triticum sativa*, *Parietaria judaica, Artemisia vulgaris*, *Plantago lanceolata, Chenopodium album*, *Salsola kali*, *Alternaria alternata*, Cat, Dog, and *Dermatophagoides pteronyssinus,* together with positive (Histamine 10 mg/mL) and negative controls. All participants were asked to stop using any antihistamines (for at least 7 days) and nasal corticosteroid sprays (for at least 2 weeks) before undergoing nNO measurement and SPTs [14,15,16].

Based on their SPT results, participants were classified in the AR and NAR groups. Anamnesis was crucial to decide whether positive SPTs were connected to symptoms, so the allergist that performed SPTs took the volunteers’ medical history, asking details beyond the questionnaire to confirm the diagnosis of AR, including questions about other possible etiologies such as hormonal rhinitis and rhinitis medicamentosa. Students were included in the AR group when they had at least one positive SPT result relevant to symptom exacerbation. Participants with negative SPTs or with positive SPTs irrelevant to symptom occurrence were classified as NAR.

We intended to examine the potentially different effects of sensitivity to perennial or seasonal allergens on nNO levels, and so participants were asked to indicate the time frames of their symptoms, and the “old-fashioned” terms of seasonal AR (SAR) and perennial allergic rhinitis (PAR) were used. SAR was defined as AR due to sensitization to seasonal allergens only, while PAR was defined as AR with sensitizations to perennial allergens, with/without seasonal allergens. To exclude biased answers, information on symptom seasonality was collected before the performance of SPTs.

The nNO measurements and SPTs were performed on the same day, during the pollen season, in April 2023.

### 2.3. Measurement of Nasal NO

Measurements of nNO were performed with the NIOX VERO portable nitric oxide analyzer (Circassia, NIOX Group PLC, Oxford, UK), following the procedure proposed by the American Thoracic Society and European Respiratory Society [17]. In order to perform the nNO measurement, the volunteer under examination was comfortably seated, thoroughly cleared the nasal passage, and was asked to indicate the nostril with an apparently better airflow. In each participant, two repeated measurements were registered (from the same nostril), with an instrument flow rate of 5 mL/s, at an interval of a minute. Each participant exhaled steadily and continuously against resistance for a total duration of 30 sec. In case the two measurements had >20% difference, a third measurement was performed. The final value registered for each subject was the mean value of the two attempts. When 3 measurements were performed, we obtained the mean value of the 3 showing the smallest deviation.

### 2.4. Statistical Analysis

The evaluation of the diagnostic value of nNO measurement for the differential diagnosis of the two groups, as well as the sensitivity and specificity of the method for different cut-offs, were calculated through receiver operating characteristic (ROC) curve analysis and the calculation of the Area Under Curve (AUC). Finally, the nNO concentration was reported as mean ± SD for each study group and the difference between the values of nNO between AR and NAR classifications was assessed using the independent samples *t*-test.

Subgroup analysis was performed within the subjects with AR to assess differences between participants with perennial vs. participants with seasonal sensitivities. The levels of nasal NO across the two groups (perennial vs. seasonal sensitivities) were assessed using independent samples t-tests in an unadjusted analysis and using an adjusted linear regression model controlling for age and gender. Within the same subgroup (AR), the relationship between the values of nNO (dependent variable) and the number of positive SPTs (independent variable) was also calculated through linear regression analysis and adjusted for age and gender, and the analysis was repeated for participants with perennial and seasonal sensitivities, respectively.

Measurements with a *p*-value of less than 0.05 were considered statistically significant. The statistical analysis of all registered data was performed using the SPSS program for Windows (IBM SPSS Statistics 27).

## 3. Results

### 3.1. Study Groups

A total of 122 students (mean age: 20.63 ± 2.37, with 65.1% females) replied to the questionnaire; 62 out of them reported symptoms of rhinitis and participated in the study. Overall, 39 had positive SPT results matching their anamnesis and were classified as AR subjects, while 23 had no SPT reactivity and were classified as NAR subjects (Table 1). Twenty AR patients were monosensitive, and nineteen had more than one positive SPT result.

Twenty participants (51.3%) were classified as SAR and nineteen (48.7%) were classified as PAR. Most SAR individuals were sensitive to O. europea and/or Graminaceae, while most PAR individuals were allergic to D. pteronyssinus. Some NAR volunteers also reported seasonal exacerbations of rhinitis. Apparently, the seasonal exacerbation of rhinitis symptoms does not always translate into SAR and conditions such as the local AR or vasomotor rhinitis may be the background of a seasonal onset of nasal symptoms in patients with negative SPT results [18,19].

### 3.2. Diagnostic Accuracy of nNO for AR

**Figure 1:**
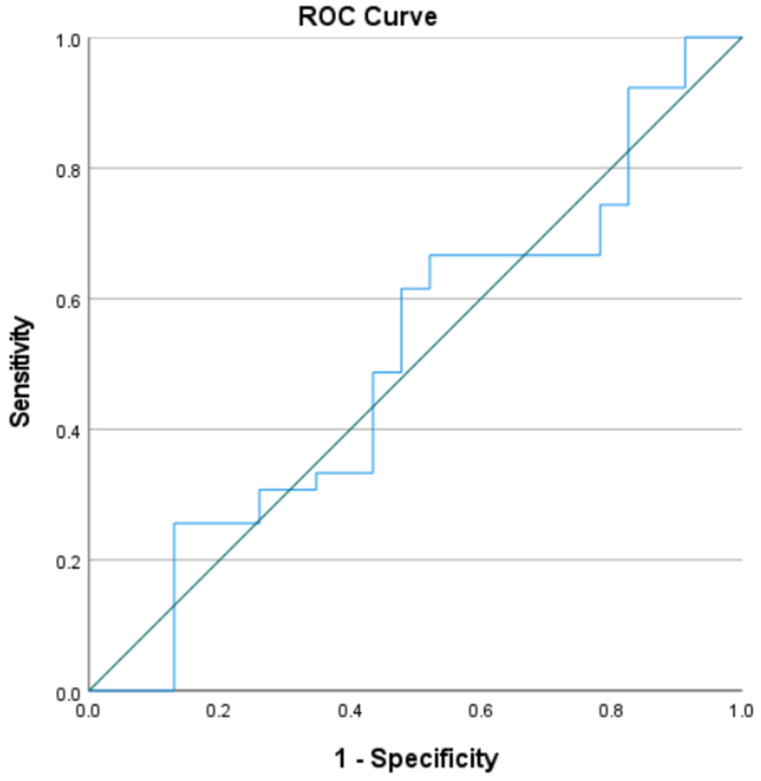
Receiver operating characteristic (ROC) curve for the differentiation of allergic rhinitis from nonallergic rhinitis by nNO measurement.

The study took place during the pollen season when nasal allergic inflammation, due to either seasonal or perennial sensitizations, should be present in the majority of the AR volunteers. Nevertheless, the mean nNO concentrations in the AR group were 830 ± 247 ppb, and we surprisingly found similar results in the NAR group, with a mean of 851 ± 373 ppb. There was no statistical difference between the two groups (*p*-value = 0.811). We calculated the diagnostic value of nNO for differentiating AR from NAR, as well as the sensitivity and specificity of the method, through an ROC curve, as illustrated in Figure 1. The ROC curve for the measurement of nNO had AUC = 0.511, suggesting that the nNO measurement’s ability to differentiate AR from NAR is comparable to that of random guessing. The best possible cut-off point given by this ROC curve analysis was 736 ppb, with 61.5% sensitivity and 52.2% specificity, confirming the inability of the nNO measurement to adequately differentiate AR from NAR.

The subgroup analysis in the AR group, revealed mean nNO concentrations of 825 ± 250 ppb and 849 ± 251 ppb for participants with PAR and SAR, respectively (*p*-value = 0.629). However, the outcome of this comparison was not surprising since the study took place during the pollen season when symptoms should be present in both AR subgroups. In the analysis controlling for age and gender, the SAR participants were associated with a 51 ppb (95%CI: −96.54, 198.99) increase in nasal NO, although this difference was not statistically significant (*p*-value = 0.486).

**Figure 2:**
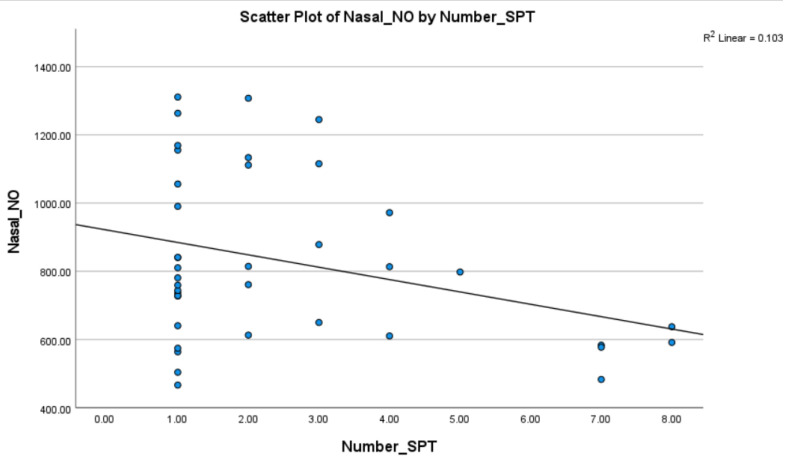
Simple linear regression between nNO levels and the number of positive SPTs.

In the analysis, examining the relationship between the number of positive SPTs and the nNO among AR patients, nNO was not significantly associated (*p*-value = 0.750) with the number of sensitizations (Figure 2), with an estimated β regression coefficient equal to -6.2 (−45.41, 32.99). The same analysis was repeated within PAR (β = −3.37, 95%CI: −68.62, 61.89, 0.915) and within SAR individuals (β = −8.97, 95%CI: −71.33, 53.39, 0.759), and also occurred without any statistically significant outcomes.

## 4. Discussion

After the initial discovery that NO has a role as an immune mediator in lower airways, it was shown that the levels of FeNO increased in asthmatics compared to control subjects [20,21]. Attempts have been made to use FeNO as a biomarker for allergic asthma [7]. The concept of “one airway, one disease”, associating AR with asthma, has inspired the study of NO in the upper airways and the evaluation of nNO levels in patients with sinonasal inflammatory diseases, including primary ciliary dyskinesia, cystic fibrosis, chronic rhinosinusitis, and AR [10,22,23]. The present study evaluated the potential use of nNO as a discriminator between AR and NAR, but denoted that it is not a biomarker for such a differential diagnosis and has no additional diagnostic value for AR.

Some prior studies have shown elevated levels nNO levels in AR patients when compared with healthy controls (HC) [15,16,24,25,26,27], but these outcomes were not confirmed by other studies [28,29,30]. A study reported significantly higher nNO levels in AR compared to HC, in HC compared to NAR, and in AR compared to NAR [13]. Likewise, in another study, higher nNO levels were demonstrated in AR patients compared to NAR ones [31]. Additionally, a study assessing nNO while considering the effect of sinus obstruction revealed significantly higher nNO levels in AR compared to HC, but no difference in levels between AR and NAR, with nNO levels in NAR being within the suggested normal range and only slightly elevated compared with HC [12]. There are various methodological factors that might explain the discrepancies between studies: the use of different analyzers and sampling techniques, the achieved trans nasal flow rates, external or confounding factors that affect levels of nNO, and the heterogeneity of study populations.

The presence of sinus inflammation without nasal polyposis has been connected to low nNO levels in both AR and NAR [13], while a negative correlation between nNO and total sinus ostial obstruction has been shown in AR patients [12]. In another study, AR patients with moderate–severe rhinitis had (non-statistically significant) lower nNO levels than those with mild [25]. The authors of this study speculate that the swelling of the nasal mucosa and secretions block the ostiomeatal complex and disturb the diffusion of NO from the paranasal sinuses [25]. Thus, performing an nNO measurement in SAR patients during the pollen season may result in lower levels, rather than higher ones, and could be a reasonable explanation for the low nNO levels in some of our participants with AR. However, the clinical examination did not reveal cases of moderate–severe symptoms, and no participant showed a seriously restricted nasal flow when performing the test.

A secondary aim of our study was to assess the association between the number of positive SPTs and the amounts of nNO in the AR group. A non-statistically significant trend of inverse correlation was observed. The probable relationship between allergy/atopy factors (such as the total IgE level, eosinophil count in peripheral blood and the wheal size of SPT) and nNO levels was examined in former studies, but they failed to demonstrate any significant correlation [25,32].

We consider that the recruitment of volunteers, instead of patients referred to an outpatient clinic, is a strength of our study. Another strength of our study is that both AR and NAR groups were examined using the same protocol and nNO measurements were “blind”, since the researcher was different from the allergist that performed SPTs and ignored the atopic status of the examined subject (SPTs were performed later). The fact that all nNO measurements were performed by the same researcher and all SPTs were performed by the same allergist excluded the chance of any subjective heterogeneity.

Pollen season in our geographical area is limited to January for *Cupressus sempervirens* and March to mid-July for grasses, *Oleaceae*, and weeds. *Alternaria* mainly triggers allergic symptoms during late summer months. Measurements of nNO were performed in April, a month considered the peak of the pollen season [33]. The fact that the examination of each volunteer was not repeated on another date and that a subject with AR might not be in his/her pollen season during the nNO measurement could be an explanation for the non-significant difference in terms of nNO between AR and NAR, and it is a limitation of our study. This might occur for *Cupressus-* and *Alternaria*-monosensitive subjects. However, we only defined an *Alternaria*-monosensitive male, and excluding these data from the analysis made no statistical difference.

Another limitation of our study was the absence of a comparison with an HC group. Comparing nNO measurements in individuals with AR to those in HC might have revealed nNO as a potential biomarker for rhinitis. The absence of an HC group was due to the fact that participants were drawn from the cohort of our PARC study—a project in which, according to the protocol, only subjects with rhinitis symptoms were invited to participate in the clinical phase that included SPTs. Furthermore, for some specific cases of nasal pathologies, such as septal deviation or unilateral rhinosinusitis, the measurement of nNO in both nostrils could have been more appropriate. Nevertheless, all participants perceived nasal airflow from either of their nostrils as normal and any differences between nostrils in the uncommon case of unilateral rhinosinusitis were unlikely to significantly influence our findings. Lastly, there was no possibility of determining if negative SPT subjects (defined as NAR) were cases of idiopathic, vasomotor, local allergic rhinitis, or nonallergic rhinitis with eosinophilia syndrome (NARES).

A nasal provocation test is a useful tool to confirm the diagnosis of AR and to perform the diagnosis of local AR. It is not a standard procedure performed in patients with negative SPT results, and so a limited number of patients with local AR may have been undiagnosed. Although this is a limitation of our study, it would theoretically only have an impact if the number of patients with local AR was high.

Immunological biomarkers may offer greater utility in the definition of AR. Notably, a recent research has indicated a correlation between invariant natural killer T (iNKT) cell subgroups and nNO levels, suggesting iNKT cells in play a role in NO-mediated AR pathogenesis [34]. This association underscores the potential of iNKT cell profiles as diagnostic markers for AR patients.

## 5. Conclusions

In conclusion, the outcome of the present study was that no significant difference was detected in nNO levels between AR and NAR. Therefore, nNO cannot be used as an AR/NAR differentiator and is not a reliable diagnostic tool that can be used in clinical practice for the diagnosis of either subtype of rhinitis.

## Figures and Tables

**Table 1:** Sensitization profiles assessed by skin prick tests among students with allergic rhinitis.

Sensitization Status	Summary Statistics
Seasonal allergens, N (% with a positive test)	
*Olea europea*	14 (35.9)
*Cupressus sempervirens*	7 (17.9)
*Phleum pratense*	8 (20.5)
*Triticum sativa*	6 (15.4)
*Zea mais*	8 (20.5)
*Parietaria judaica*	4 (10.2)
*Artemisia vulgaris*	6 (15.4)
*Plantago lanceolata*	6 (15.4)
*Chenopodium album*	3 (7.7)
*Salsola kali*	5 (12.8)
*Alternaria alternata*	4 (10.2)
Perennial allergens, N (% with a positive test)	
Cat	8 (20.5)
Dog	1 (2.5)
*Dermatophagoides pteronyssinus*	18 (46.1)
Number of sensitizations; median (range)	2 (1-8)

## Data Availability

The data presented in this study are available on request from the corresponding author due to 31 August 2025.
